# FBXW7 and USP7 regulate CCDC6 turnover during the cell cycle and affect cancer drugs susceptibility in NSCLC

**DOI:** 10.18632/oncotarget.3708

**Published:** 2015-03-30

**Authors:** Francesco Morra, Chiara Luise, Francesco Merolla, Ina Poser, Roberta Visconti, Gennaro Ilardi, Simona Paladino, Hiroyuki Inuzuka, Gianluca Guggino, Roberto Monaco, David Colecchia, Guglielmo Monaco, Aniello Cerrato, Mario Chiariello, Krista Denning, Pier Paolo Claudio, Stefania Staibano, Angela Celetti

**Affiliations:** ^1^ Istituto per l'Endocrinologia e l'Oncologia Sperimentale “Gaetano Salvatore”, CNR, Napoli, Italy; ^2^ Dipartimento di Medicina Molecolare e Biotecnologie Mediche, Università Federico II, Napoli, Italy; ^3^ Dipartimento di Scienze Biomediche Avanzate, Università Federico II, Napoli, Italy; ^4^ Max Plank Institute, MPI-CBG Dresden, Germany; ^5^ Harvard Medical School, Beth Israel Deaconess Medical Center, MA, USA; ^6^ UOC Chirurgia Toracica, Azienda Ospedaliera di Rilievo Nazionale “A.Cardarelli”, Napoli, Italy; ^7^ UOC Anatomia Patologica, Azienda Ospedaliera di Rilievo Nazionale “A.Cardarelli”, Napoli, Italy; ^8^ Istituto Toscano Tumori, Core Research Laboratory, Siena, Italy; ^9^ Department of Pathology, Joan C. Edwards Cancer Center, Huntington, WV, USA; ^10^ Department of Biochemistry and Microbiology & Dept. of Surgery, Marshall University, Joan C. Edwards Cancer Center, Huntington, WV, USA

**Keywords:** CCDC6, FBXW7, USP7, mitotic kinases, cisplatinum

## Abstract

CCDC6 gene product is a pro-apoptotic protein substrate of ATM, whose loss or inactivation enhances tumour progression. In primary tumours, the impaired function of CCDC6 protein has been ascribed to CCDC6 rearrangements and to somatic mutations in several neoplasia. Recently, low levels of CCDC6 protein, in NSCLC, have been correlated with tumor prognosis. However, the mechanisms responsible for the variable levels of CCDC6 in primary tumors have not been described yet.

We show that CCDC6 turnover is regulated in a cell cycle dependent manner. CCDC6 undergoes a cyclic variation in the phosphorylated status and in protein levels that peak at G2 and decrease in mitosis. The reduced stability of CCDC6 in the M phase is dependent on mitotic kinases and on degron motifs that are present in CCDC6 and direct the recruitment of CCDC6 to the FBXW7 E3 Ubl. The de-ubiquitinase enzyme USP7 appears responsible of the fine tuning of the CCDC6 stability, affecting cells behaviour and drug response.

Thus, we propose that the amount of CCDC6 protein in primary tumors, as reported in lung, may depend on the impairment of the CCDC6 turnover due to altered protein-protein interaction and post-translational modifications and may be critical in optimizing personalized therapy.

## INTRODUCTION

To cope with the genotoxic damage, cells activate powerful DNA damage induced cell cycle checkpoints that coordinate cell cycle arrest with recruitment and activation of the DNA repair machinery [1-5]. The global relevance of the cell cycle checkpoint pathways in maintaining genomic integrity is highlighted by the observation that loss, inactivation, or epigenetic silencing of checkpoint genes is frequently observed in cancer. Deletion of checkpoint genes has been shown to cause genomic instability and predisposition to transformation [6, 3]. Indeed, loss of DNA damage checkpoints, during early stages of tumorigenesis, may facilitate the acquisition of additional mutations over the time, determining aggressiveness and drug response in tumors.

In previous work we documented that CCDC6 acts as a pro-apoptotic protein substrate of ATM, to sustain DNA damage checkpoints in response to genotoxic events [7, 8]. Moreover, CCDC6 protects genome integrity by modulating the activity of the phosphatase PP4C directed towards the de-phosphorylation on S139 of the histone H2AX (γH2AX) in response to DNA damage [9]. Consistent with these observations, we reported that CCDC6 deficiency affects the γH2AX foci formation and the repair of the DNA DSBs [9]. Thus, CCDC6 is an attractive candidate biomarker whose loss or inactivation could enhance tumor progression by impairing apoptosis thereby favoring cell survival and allowing the cells to overcome the barrier of a DNA damage response. The CCDC6 gene was originally identified because of its rearrangement with the RET tyrosine kinase in thyroid tumors [10], and with genes other than RET in non-solid tumors [11]. In most cancers that harbor the CCDC6 gene rearrangements, the product of the normal allele of CCDC6 is either wholly absent or functionally impaired by a dominant negative mechanism [7]. Moreover, CCDC6 sporadic mutations have been reported in ovary and large intestine tumors (www.sanger.ac.uk/genetics/CGP/cosmic). Recently, CCDC6-RET fusions and CCDC6 point mutations [N394Y, T462A, S351Y, E227K] have been reported in NSCLC [12, 13]. Defective expression of CCDC6 has been observed in about 30% of NSCLC and negatively correlated to DFS and OS [14]. Importantly, in NSCLC cells, the defective expression of CCDC6 causes an impaired DNA repair by homologous recombination (HR), making these cells sensitive to PARP inhibitors. The concurrent treatment of cisplatinum and PARP inhibitors has shown a synergistic effect in CCDC6 deficient NSCLC cells [14].

CCDC6 has been recently recognized as a novel target of the E3 ubiquitin ligase FBXW7 for ubiquitin-mediated proteasomal degradation [15].

The ubiquitin-mediated protein degradation pathway plays an important role in controlling the abundance of intracellular proteins and plays an essential role in maintaining normal cellular function, including the cell cycle. Deregulation of ubiquitin-mediated proteolysis results in the development of a variety of human cancers [16].

The removal of ubiquitin conjugates from target protein by de-ubiquitylating enzymes (DUBs) has emerged as an important regulatory mechanism in a range of cellular processes [17]. DUBs activity may be considered as important as the E3-ubiquitin ligases in cancer development, underscoring the dynamic and reversible nature of protein ubiquitylation. Several DUBs have been found to function in the DNA damage response, including ubiquitin-specific protease 1 (USP1), USP7 and USP28 [18, 19]. USP7 (also known as HAUSP) is a DUB for several tumor suppressors [20]. Lately, USP7 has been predicted as a major DUB for CCDC6 [21]. Here, we investigated if USP7 is specifically able to interact with CCDC6, controlling the CCDC6 stability and counteracting the FBXW7-mediated ubiquitylation of the protein.

In this work we have analysed the post-translational modifications and the mechanisms regulating CCDC6 protein turnover during cell cycle progression in order to establish whether the increased degradation or the reduced stability of CCDC6 might drive a different sensitivity to PARP inhibitors in cancer cells.

## RESULTS

### CCDC6 levels are regulated in a cell cycle dependent manner by phosphorylation events

**Figure 1:**
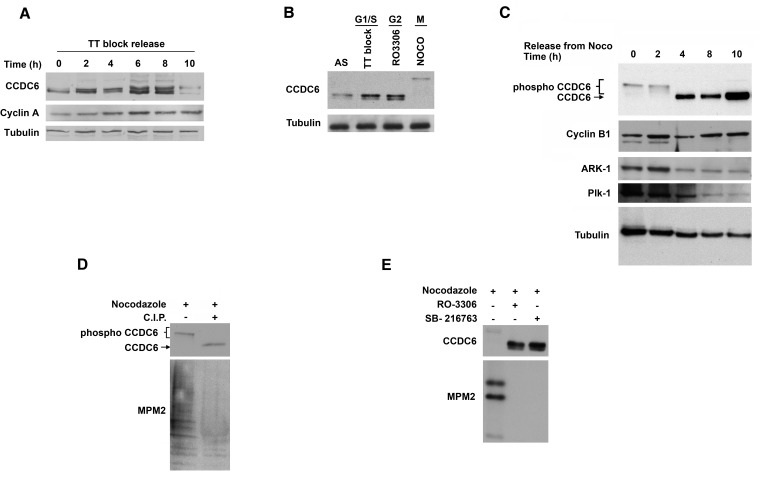
CCDC6 protein levels are regulated through the cell cycle **A**) CCDC6 levels increase from G1 to mitosis. Human HeLa cells were synchronized at the G1/S boundary by double thymidine block (TT block), then released into the cell cycle. Samples were analysed by SDS-PAGE and immunoblotted using the specific antibodies at the times shown after release from the block. **B**) HeLa cells were synchronized at the G1/S boundary by TT block, at the G2 phase by the CDK1 inhibitor RO3306, in mitosis after treatment with 100 ng/ml nocodazole (Noco) for 16 hours. Floating cells were separated from adherent and samples were analysed by immunoblot using the anti-CCDC6 antibody. **C**) Human HeLa cells were treated with 100 ng/ml nocodazole for 16 hours. Floating, mitotic cells, were separated from adherent (interphase) cells, plated again and samples were taken at the indicated times and analysed by immunoblotting using the indicated antibodies. **D**) HeLa cells were synchronized as in C and collected at time 0. Extracts were treated *in vitro* with CIP, as indicated. Therefore, samples were taken and analysed by immunoblotting with the indicated antibodies. Anti-MPM2 is utilized as indicator of mitotic arrest. **E**) HeLa cells were synchronized as in C, and cells were treated with RO3306 (9 μM for 2 hours) or with SB216763 (10 μM for 4 hours) before the nocodazole release, as indicated. Samples were analysed by SDS-PAGE and immunoblotted using the indicated antibodies.

To investigate the regulation of CCDC6 protein levels during the cell cycle, human HeLa cells were synchronised at the G1/S boundary using a double thymidine (TT) block, then released into the cell cycle. The time course experiment showed several post-translational modifications of CCDC6 protein at different time points (Figure 1A). In order to better discern the CCDC6 behaviour during the cell cycle, we utilized different chemical treatments to block the cells in specific phases. We observed a cyclic variation of CCDC6 levels that increased at boundary G1/S, picked at G2, and appeared upshifted in mitotic phase (Figure 1B). In order to focus on the CCDC6 modifications in mitosis, HeLa cells were then treated with nocodazole for 16 h, collected by shake-off, plated again and analyzed at different time points for a mitotic time course. The product of CCDC6 appeared as multiple bands on SDS-page gels at time 0, from the nocodazole release. The shifts were maintained up to 2 hours, while they disappeared at 4, 8 and 10 hours when the protein seemed to be stabilized (Figure 1C). Calf intestinal phosphatase (CIP) treatment determined a significant decrease of the shifted bands at time 0, thus suggesting that the modifications observed in mitosis might depend on phosphorylation events (Figure 1D). The specific inhibition of cyclin–dependent kinase 1 (CDK1) by RO3306 reverted most of the shifts observed by electrophoresis. In addition, the pretreatment with the specific inhibitor of glycogen synthase kinase 3 (GSK3), SB216763, also reverted the modifications detected for CCDC6 in mitosis (Figure 1E).

**Figure 2:**
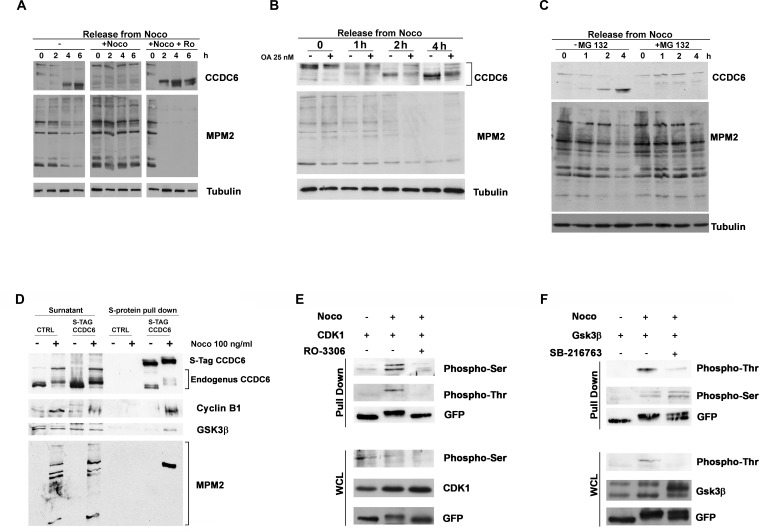
CCDC6 behaviour during mitotic arrest depends on the CDK1 activity **A**) HeLa cells were treated as in (1C). RO3306 and nocodazole treatment were maintained for additional 6 hours, before sampling and analysis by immunoblot, as indicated. **B**) HeLa cells were synchronized as in 1C, in presence or absence of Okadaic Acid (25 nM, one hour before arrest in mitosis) collected at the indicated times and analysed by immunoblotting using the indicated antibodies. **C**) Cells were treated with MG132 (10 μM) for 2 hours before arrest in mitosis as in (1C) and maintained in MG132 for additional 4 hours. Samples were immunoblotted with the antibodies shown. **D**) S-tag Pull Down of HeLa-Kyoto S-tag-GFP-CCDC6 asynchronous or mitotic extracts were analysed by SDS-PAGE and immunoblotted with the anti-cyclin B and anti-GSK3 antibodies, as shown. The anti-CCDC6 hybridization detected the S-tag-CCDC6 and the endogenous CCDC6, as indicated. The proteins expression in the surnatant is shown on the left side of the immunoblot. **E**) **F**) S-tag Pull Down of HeLa-Kyoto S-tag-GFP-CCDC6 asynchronous or mitotic extracts from cells overexpressing CDK1 (E) or GSK3 (F) constructs previously treated with RO3306 at 9 μM for 2 hours or with SB216763 at 10 μM for 4 hours, respectively, before arrest in mitosis, as indicated, were analysed by SDS-PAGE and immunoblotted with the specific antibodies, as shown. The immunoblots of the whole cell lysates (WCL) are shown at the bottom of the panels E and F, respectively.

We maintained the CCDC6 mitotic phosphorylation status by keeping the cells in nocodazole for additional 2, 4 and 6 hours, after a pretreatment of 16 hours. The addition of the CDK1 inhibitor RO3306, during the nocodazole maintenance, impeded the CCDC6 post-translational modifications that occurred in mitosis, suggesting that CCDC6 is kept in the phosphorylated status mainly by CDK1 (Figure 2A). At 2 and 4 hours from nocodazole release the non-phosphorylated status of CCDC6 was mildly reverted by the okadaic acid addition suggesting that the activity of the mitotic kinases keeps the CCDC6 phosphorylation status in mitosis as well as phosphatases contribute to regulate the CCDC6 phosphorylation status at mitotic exit (Figure 2B). In mitotic cells, treated with the proteasome inhibitor, MG132 (up to 4 hours), CCDC6 shows a reduced mobility on SDS-PAGE suggesting that in these conditions CCDC6 is stuck in a phosphorylated status (Figure 2C). The MG132 treatment causes a reduced degradation of cyclin B1 that maintain CDK1 active on newly synthetized CCDC6 [22].

### CCDC6 gene product binds CDK1 and GSK3 mitotic kinases

We wanted to investigate if CCDC6 was able to interact with the mitotic kinases, whose inhibitors reverted the CCDC6 phosphorylation observed in mitosis. To this aim we performed a S-protein pull-down in mitotic HeLa Kyoto cells, stably expressing S-tag-GFP-CCDC6 construct [23]. By this experiment we identified a specific interaction between CCDC6 and endogenous cyclin B1, a component of the CDK1-cyclinB complex. Moreover, the mitotic pull down showed that CCDC6 was also able to interact with the GSK3 kinase (Figure 2D). Interestingly, the endogenous CCDC6, that is likely to be pulled down by the heterodimerization with the S-tag-CCDC6 protein, appeared to be shifted as well on the gel in the mitotic lysate. Moreover, the hybridization with the phospho-antibody anti-MPM2, originally cloned on the basis of its ability to recognize mitotic phosphorylated residues on Cdk1/2 consensus motifs [22, 24-26], clearly showed immunoreactivity for CCDC6 in a pull down performed on mitotic extracts (Figure 2D). CCDC6 truncated mutants [(1-101) and (1-223)] did not show CCDC6 protein shifts after nocodazole release, suggesting that the target residues of the mitotic kinases may be located downstream of the aminoacid 223 in the CCDC6 sequence (Supplementary Figure 1). Several Serine/Threonine consensus for both CDK1 and GSK3 mitotic kinases (http://scansite.mit.edu) have been predicted in the CCDC6 protein sequence. We performed a CCDC6 pull-down in mitotic extracts from HeLa Kyoto S-tag-GFP-CCDC6 cells overexpressing CDK1 or GSK3. In pull-down of cells overexpressing CDK1 we detected a predominant immunoreactivity for phosphoserine residues of CCDC6, while in pull-down from cells overexpressing GSK3 kinase we observed a predominant immunoreactivity of the phosphothreonine residues of CCDC6. By treating the cells with RO3306 or SB216763 we were able to impede prevalently the recognition of CCDC6 phosphorylated residues, respectively, by antiphosphoserine and the antiphosphothreonine antibodies. These data support the idea that in mitosis the phosphoserine residues of CCDC6 are predominantly substrates of CDK1, while the phosphothreonine residues are predominantly substrates of GSK3 (Figure 2E and 2F).

### Mutations of the phosphoresidues in the CCDC6 degron motifs for FBXW7 recognition sites affect CCDC6 cell cycle regulated turnover

**Figure 3:**
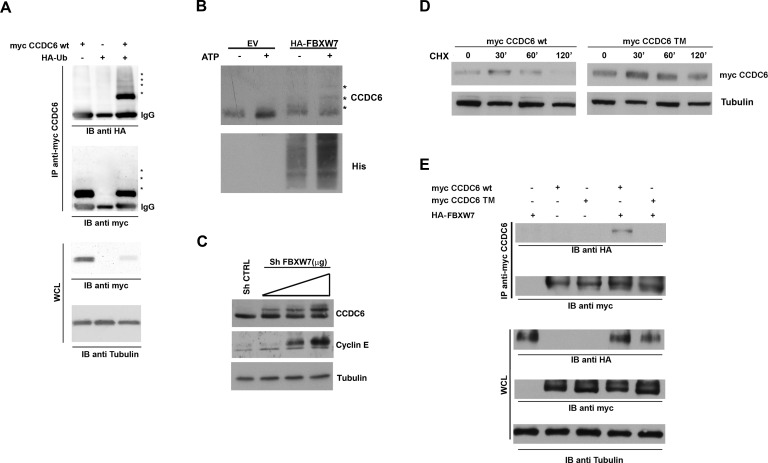
FBXW7 increases CCDC6 turnover via increasing CCDC6 ubiquitination **A**) 293T cells were transfected with the indicated plasmids. The amount of ubiquitinated CCDC6 was analysed by immunoprecipitation (IP) of myc-CCDC6 followed by immunoblotting (IB) with anti-HA. **B**) Affinity-purified GST-CCDC6 recombinant protein (Abnova H00008030-P01) was incubated with purified E1, E2, His-ubiquitin and 293T cell lysate as source of E3 ligase FBXW7, from cells transiently transfected with HA-FBXW7, at 37°C for 60 min (according to manufacturer instructions; see M&M). The ubiquitination reaction products were resolved by SDS-PAGE and probed with anti-Histidine antibody and anti-CCDC6 antibody. **C**) Sh-FBXW7 constructs, at 2, 3 or 4 μg doses, were transiently transfected in 293T cells. Cell lysates were immunoblotted with the indicated antibodies. Expression levels of cyclin E are shown as a representative substrate of FBXW7. **D**) CCDC6 turnover rate is reduced in HeLa cells overexpressing the mutated triple mutant CCDC6 construct (SST359/413/427AAA) (TM), compared to the CCDC6 wild type, (WT), construct overexpressing cells. Cells were treated with cycloheximide (CHX) (100 ng/ml) for the indicated times. Cell lysates were immunoblotted with the indicated antibodies. **E**) Myc-CCDC6 WT and TM constructs were cotransfected with HA-FBXW7 into 293T cells in presence of MG132 (25 μM). Cell lysates were immunoprecipitated with anti-myc and immunoblotted with the anti-HA antibody. Whole cell lysates, shown at the bottom of the figure, have been immunoblotted with the indicated antibodies.

Proper phosphorylation of protein substrates is required for FBXW7 recognition and targeting for degradation [27]. Recently, CCDC6 has been recognised as a novel FBXW7 E3 ubiquitin ligase (Ubl) substrate [15]. In order to determine if CCDC6 was modified by HA-Ubiquitin, we performed an immunoprecipitation assay with myc-CCDC6 construct and we detected CCDC6 modifications, reminiscent of ubiquitinylation (Figure 3A). We next determined that recombinant CCDC6 is directly ubiquitylated by the reconstituted FBXW7-containing Skip1-Cullin-Fbox (SCF) complex, by an *in vitro* assay (Figure 3B). Interestingly, the incubation with CDK1 enhanced the FBXW7-mediated ubiquitination of the recombinant GST-CCDC6, in support of the primary role exerted by this kinase (Supplementary Figure 2). Furthermore, we observed that transient silencing of FBXW7 in HeLa cells was able to stabilize the CCDC6 protein in a dose dependent manner, *in vivo* (Figure 3C).

**Figure 4:**
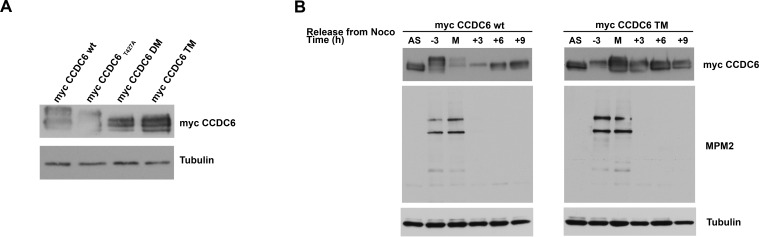
CCDC6 turnover is impaired by mutations in the phosphoresidues of the CCDC6 degron motifs recognized by FBXW7 **A**) Assessment of the stability of the CCDC6 mutant proteins [single (S359A), double (ST359/427AA) and triple (SST359/413/427AAA) mutants] by western blotting at mitotic arrest, after 16 hours nocodazole (100ng/ml) and hybridization with the indicated antibodies. **B**) Assessment of CCDC6 TM levels by western blot during mitosis. The mitotic time course indicates when synchronized cells were collected on relation to the onset of mitotic arrest, that is, −3 denotes 3 hours before mitosis (M) and +3 denotes 3 hours after cells entered mitosis and beyond (+6, +9). Anti-MPM2 is utilized as indicator of mitotic arrest. Anti-tubulin is indicator of equal loading.

In CCDC6 protein sequence, the residues serine 359, serine 413 and threonine 427 have been recognized as canonical FBXW7 degron motifs and conserved across different species [15]. A CCDC6 mutant in the mentioned residues showed an increased half life when overexpressed in HeLa cells in presence of cycloheximide (CHX), an inhibitor of *de novo* protein synthesis (Figure 3D). Moreover, in experiment of co-immunoprecipitation we have observed that the CCDC6 triple mutant is impaired in its binding to FBXW7 (Figure 3E). Thus, the data produced by us and by others [15] suggest that the phosphorylation of the CCDC6 residues, that are targets of GSK3, allows FBXW7 to act as a negative regulator of CCDC6 stability. In order to define if the degradation of CCDC6 in mitosis is dependent on FBXW7 binding upon phosphorylation of the GSK3 target residues (S359, S413, T427), we expressed in HeLa cells the single, S359A, double, ST359/427AA (DM), and triple, SST359/413/427AAA (TM) mutants of CCDC6 that showed a defect in the mitotic shifts after 16 hours of treatment with nocodazole (time 0 from nocodazole release) with a progressive reduction that appeared related to the number of the modified residues, with a striking protein stabilization observed in the triple mutant (Figure 4A). Moreover, the overexpression of myc-CCDC6 constructs, mutated in all the phosphodegron recognition sites (triple mutant, TM), did not show the cyclic variation observed for the myc-CCDC6 wt protein during the mitotic time course (Figure 4B). Therefore, mutations in the CCDC6 phosphoresidues, recognized by GSK3, are responsible of the impaired turnover of CCDC6 during the progression of the cell cycle.

### FBXW7 targets CCDC6 for proteasomal degradation during mitotic arrest

**Figure 5:**
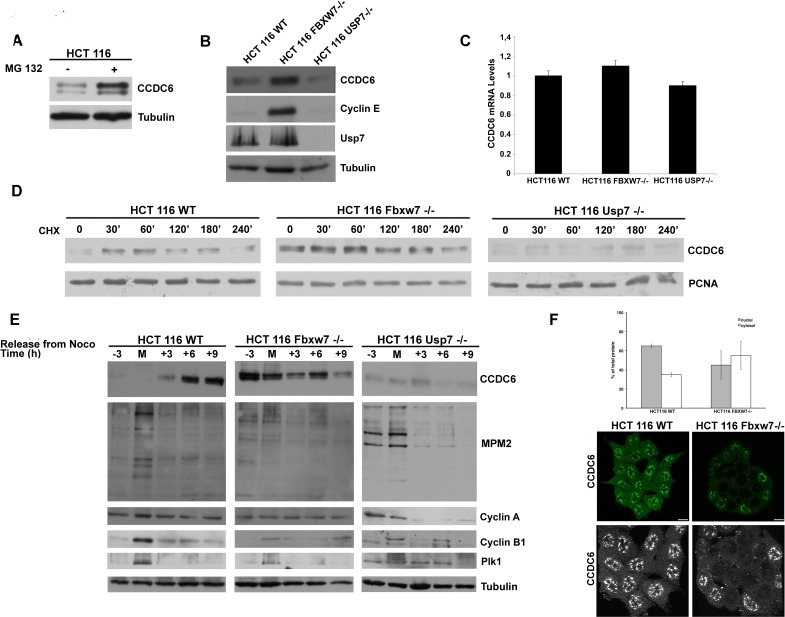
FBXW7 deficiency leads to CCDC6 stabilization and affects CCDC6 turnover rate **A**) HCT116 wild type cells were treated with MG132 (20 μM) for 6 hours before harvesting. Equal amounts of cell lysates were immunoblotted with anti-CCDC6 or anti-tubulin antibodies. **B**) Indicated wild type, FBXW7−/− and USP7−/− cells were harvested and equal amounts of cell lysates were immunoblotted with anti-CCDC6, cyclin E, USP7 or tubulin antibodies. **C**) CCDC6 relative mRNA expression by quantitative RT-PCR in human HCT116 WT, FBXW7−/− and USP7−/− cells. **D**) CCDC6 turnover rate is delayed in FBXW7 deficient cells and accelerated in USP7 deficient cells. Indicated wild type (WT), FBXW7−/− and USP7−/− HCT116 cells were treated with cycloheximide (CHX) (100 ng/ml) for the indicated times. Cell lysates were immunoblotted with the indicated antibodies. **E**) Assessment of CCDC6 levels by western blot during mitotic time course as in (4B) in HCT116 WT, FBXW7−/− and USP7−/−. Anti-MPM2, Cyclin A, Cyclin B and Plk1 have been utilized as indicators of mitotic arrest. **F**) Localization of CCDC6 by immunofluorescence assay in HCT116 WT and FBXW7−/− cells. After fixation, cells were permeabilized and stained with anti-CCDC6 antibody. Serial confocal sections were collected. Bars, 11 μm. The 3D reconstruction is shown (lower panels). Results from two different experiments were plotted as percent nuclei (grey) and cytosol (white) localization (n > 100 cells). Error bars, SD; p < 2e_11.

To further study the role of E3-Ubl FBXW7 in the regulation of endogenous CCDC6 protein during the cell cycle we employed the HCT116 FBXW7−/− colon cancer cells [28]. First, we observed that the regulation of CCDC6 stability was found to be proteasome-dependent in HCT116 wt cells, as MG132 treatment leads to stabilization of endogenous CCDC6 (Figure 5A). Moreover, we report that the HCT116 FBXW7−/− have increased levels of endogenous CCDC6 at western blot when compared with HCT116 wild type cells (Figure 5B). The impairment of the FBXW7 activity in HCT116 FBXW7−/− is confirmed by the cyclin E stabilization at western blot, that is not detected in wild type HCT116 cells [29]. Nevertheless, the CCDC6 mRNA levels were substantially constant in these cells (Figure 5C). In experiments performed with the cycloheximide we observed a delayed CCDC6 turnover (nearly 60 minutes) in the HCT116 FBXW7−/− cells compared to the HCT116 wt cells (Figure 5D). To further analyse the role of FBXW7 in the regulation of the CCDC6 turnover in mitosis, the HCT116 cells (wt and FBXW7−/−) were synchronized and the protein extracts collected throughout the mitotic time course. During mitotic arrest, CCDC6 degradation was attenuated in HCT116 FBXW7−/− cells, compared to the wild type cells (Figure 5E), indicating that the fluctuations of endogenous levels of CCDC6 protein are mostly dependent on the FBXW7 activity in mitosis. Interestingly, in HCT116 FBXW7−/− the stabilized CCDC6 protein appeared mainly localized in the cytosol (Figure 5F), a condition that has been related to tumor growth [30, 31].

### The de-ubiquitinase USP7 stabilizes CCDC6 protein

**Figure 6:**
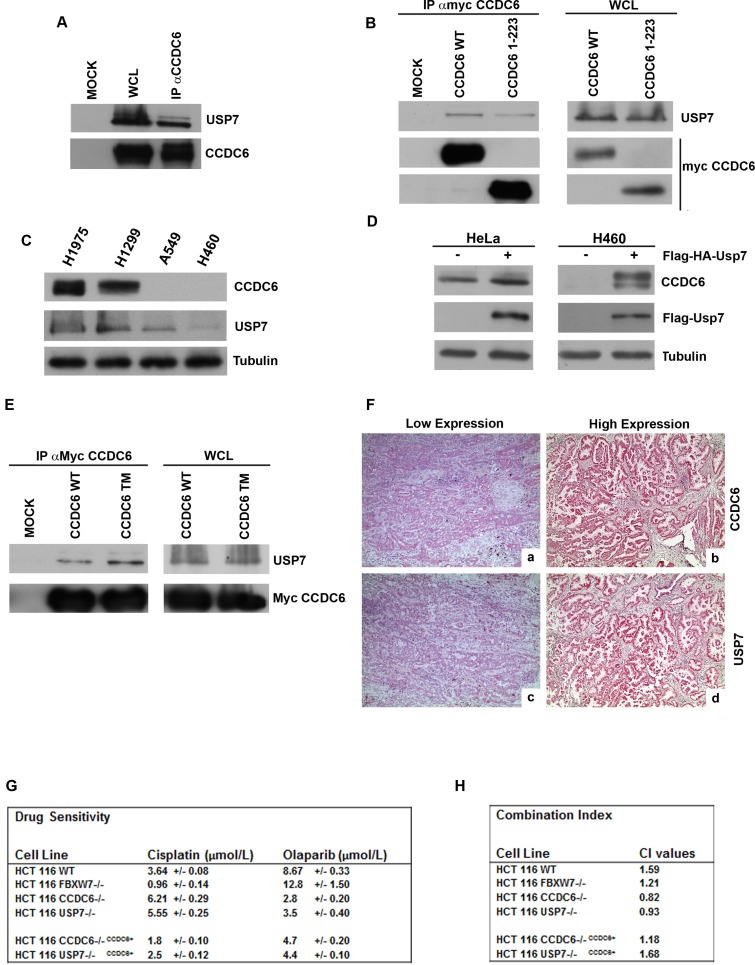
CCDC6 interacts with USP7 **A**) Endogenous CCDC6 and USP7 proteins obtained from parental 293T cells were coimmunoprecipitated. The immunocomplexes were analysed by western blotting using the indicated antibodies. **B**) CCDC6 wt or the CCDC6 (1-223) deleted mutant constructs, transfected in 293T cells, were immunoprecipitated with anti-myc. Then, the immunocomplexes were analysed by western blot using the indicated antibodies. **C**) Immunoblot analysis of USP7 and of CCDC6 in NSCLC-derived cell lines: ADC derived cell lines (NCI-H1975, NCI-H460 and A549) and Large Cell Carcinoma cell line (NCI-H1299). Antitubulin is shown as loading control. **D**) Immunoblot analysis of CCDC6 expression in HeLa cells and in H460 cells overexpressing (+) FLAG-HA-USP7. Anti-FLAG immunoblot shows the USP7 transfection efficiency. Antitubulin is shown as loading control. **E**) 293T cells were transfected with myc-CCDC6 WT and TM constructs. Whole cell lysates (WCL) were prepared and equal amounts of proteins were immunoprecipitated with anti-myc. Then, the immunocomplexes were analysed by western blot using the indicated antibodies. **F**) Immunohistochemical analyses of human lung tumor tissues. Formalin fixed paraffin embedded lung tissue sections were stained with antibody against CCDC6 (a,b) or USP7 (c,d). Representative examples are shown of: CCDC6 low expression pattern (a), CCDC6 high expression pattern (b), USP7 low expression pattern (c), USP7 high expression pattern (d). Magnification: a, b, c, d x 200. **G**) Drug sensitivity to cisplatinum and olaparib in HCT116wt, FBXW7−/−, CCDC6−/−, USP7−/−, CCDC6−/−^CCDC6+^, and USP7−/−^CCDC6+^ was determined by a modified 3-(4,5-dimethylthiazole-2-yl)-2-5-diphenyltetrazolium bromide assay, [CellTiter 96 AQueous One Solution assay (Promega)], as 50% inhibitory concentration (IC50) values. **H**) Combination index according to 1:2 concentration ratios of cisplatin and olaparib in HCT116 wt, FBXW7−/−, CCDC6−/−, USP7−/−, CCDC6−/− ^CCDC6+^, USP7−/− ^CCDC6+^ cells are shown. CI < 1, CI = 1 and CI > 1 indicate synergism, additive effect and antagonism, respectively.

USP7 is the de-ubiquitinase (DUB) predicted to remove ubiquitin from CCDC6 [21]. We verified that endogenous CCDC6 interacts specifically with USP7 (Figure 6A). The myc-tagged CCDC6 truncated mutant (1-223) still coimmunoprecipitated with USP7, suggesting that the aminoterminus of CCDC6 is sufficient for the CCDC6 binding to USP7 (Figure 6B). Recent findings unveiled that in NSCLC cell lines, H1975, H1299, A549 and H460, CCDC6 transcripts did not show significant variability, while quite different CCDC6 protein levels were observed [14]. Moreover, by cycloheximide experiments differences of the CCDC6 half life were detected in the NSCLC cells (Supplementary Figure 3).

Thus, in the lung cellular system, the H1975 and the H1299 cells, characterized by high levels of USP7 protein, show more CCDC6 product compared to the H460 and the A549 NSCLC cells that expresse less USP7 and are almost deficient for CCDC6 protein (Figure 6C). By modulating the USP7 intracellular amount, we were able to alter the levels of CCDC6 protein in different cell types. The overexpression of FLAG-HA-USP7 in HeLa and in H460 cells increased the amount of CCDC6 protein (Figure 6D). The knockdown of USP7 in HCT116 cells [32] decreased CCDC6 protein (Figure 5B), without affecting the CCDC6 mRNA, as shown in the Figure 5C. The knockdown of USP7 in HCT116 altered the CCDC6 half life, affecting its stability (Figure 5D). In order to determine the effects of the de-ubiquitinase USP7 on the CCDC6 turnover, we evaluated the amount of CCDC6 at different time point in a mitotic time course performed in HCT116 cells that were deficient for USP7. In these cells, the impairment of the CCDC6 stability is mostly evident at 6 and 9 hours from nocodazole release (Figure 5E). In general, we observed reduction of CCDC6 protein after USP7 knockdown, consistently with the fact that ubiquitin is not removed any longer from CCDC6. In an *in vitro* ubiquitination assay we observed that the ubiquitination mediated by FBXW7 complex on the recombinant CCDC6, as shown in the Figure 3B, is impaired by the presence of USP7 (Supplementary Figure 4). We also observed that alanine substitutions of the mentioned S/T residues enhanced the interaction of CCDC6 with USP7 (Figure 6E). Thus, we hypothesized that CCDC6 phosphorylation at the residues that are targets of CDK1 and GSK3 may limit the CCDC6-USP7 interaction. Interestingly, the immunostaining of 62 primary NSCLC showed that expression levels of USP7 directly correlated to CCDC6 protein levels (Figure 6F). The correlation of CCDC6 and USP7 positivity staining intensity was proved to be statistical significant (χ^2^ *test*: p ≤ 0,05) in the analyzed samples.

Recently, we have reported that the H460 NSCL cancer cells, that show low levels of CCDC6 protein, are sensitive to the PARP inhibitor olaparib, whereas H1975 cells, harboring high levels of CCDC6 protein, are resistant to olaparib and become sensitive when CCDC6 is knocked down [14]. Here we show that the knockdown of USP7 reduces the levels of CCDC6 in HCT116 cells (Figure 5B) and increases their sensitivity to olaparib (Figure 6G and 6H). Moreover, the combination of cisplatinum and olaparib has a synergistic effect on these cells. In HCT116 USP7−/− and in HCT116 CCDC6−/−, that we have generated (Supplementary Figure 5A), the reconstitution of CCDC6 is sufficient to revert the olaparib sensitivity, suggesting that CCDC6 levels are the critical determinant for the olaparib sensitivity (Figure 6G and 6H; Supplementary Figure 5B). Therefore, we suggest that the clinical usage of USP7 inhibitors [33, 34], by downregulating the CCDC6 levels, might increase the sensitization to olaparib and enhance the response to standard chemotherapeutics in combined therapy in cancer cells.

## DISCUSSION

In this study we report for the first time that CCDC6 is a cell cycle regulated protein in mammalian cells. CCDC6 levels increase at boundary G1/S, pick at G2 and decrease in mitosis; the same evidences observed in Xenopus laevis extracts support the notion that CCDC6 behaviour is highly conserved (Supplementary Figure 6). The CCDC6 modifications detected in cell cycle are mostly dependent on phosphorylation events generated by multiple cell cycle dependent kinases that prime CCDC6 for degradation in mitosis in a proteasome dependent manner.

As the control of CCDC6 stability involves both ubiquitylation and deubiquitylation processes we have characterized FBXW7 as the E3 Ubiquitin ligase responsible for the CCDC6 degradation and USP7 as the de-ubiquitynating enzyme that counteracts the FBXW7 activity for CCDC6 stabilization. The stability of CCDC6 in G2, also observed in previous work, has pointed out to the role of CCDC6 in the G2/M checkpoint [9]. Here, we report a critical role of FBXW7 in the control of CCDC6 homeostasis in mitosis by regulating its ubiquitin-proteasomal degradation and providing evidences that the CCDC6 post-translational regulation in the cell cycle may account for differences in cancer cells drug response.

Recent findings unveiled that CCDC6 different levels in NSCLC are probably due to variations in protein stability and/or turnover, since CCDC6 transcript did not show significant variability in NSCLC cell lines and NSCL FFPE primary samples [14].

Thus, we hypothesized that the alteration of CCDC6 steady-state, controlled by FBXW7 or USP7, may help to select tumors that carry FBXW7 loss/mutation or USP7 mutations, as potentially responsive to olaparib treatment. Our study shows that the FBXW7 and USP7 enzymes keep CCDC6 protein in check of G2/M phase during the cell cycle. In tumors overexpressing USP7, or carrying an impaired FBXW7-mediated degradation, CCDC6 is highly stabilized. Conversely, tumors harbouring barely detectable levels of USP7 have reduced amount of CCDC6 protein. We recently reported that defective CCDC6 drives a PARP inhibitors sensitivity in NSCLC. Thus, the identification of NSCL tumors that are deficient in USP7 may provide insight into the selection of patients that could benefit of the PARP inhibitors in combination to the conventional chemotherapy. Thus, we wonder if the use of USP7 inhibitors, by reducing CCDC6 stability, may increase the PARP inhibitors sensitivity and improve the management of NSCLC patients.

FBXW7 is considered an haploinsufficient tumor suppressor that targets several proto-oncoproteins for degradation, including cyclin E, Myc, c-jun, Mcl1 [27, 35-43]. Here, we enroll CCDC6, a protein of the DNA Damage response, as a novel FBXW7 substrate, suggesting that patients carrying FBXW7 mutations might present a more stable CCDC6 protein being more resistant to olaparib combination for their treatment. However, our data show that cells null for FBXW7 mainly maintain a cytosolic localization of CCDC6. As we have previously reported, SUMO2 post-translational modifications of CCDC6, occurring when ubiquitynation is impaired, mainly localize CCDC6 in the cytosol limiting the CCDC6 nuclear activity [30-31]. In our system we have identified a correlation between the genetic status of FBXW7 and the sensitivity to cisplatinum, as the FBXW7 null cells, in which CCDC6 is more stable, are highly responsive to platinum salts. Furthermore, our observations suggest that CDK1 possibly primes CCDC6 in order to favour GSK3 phosphorylation in the degron motifs and promotes its binding to FBXW7 in a cellular context. As well the phosphorylation events might also affect the USP7 binding in mitosis, since the CCDC6-USP7 interaction appears to be stronger during the cellular interphase, when CCDC6 is more stable (Supplementary Figure 7). Our model suggest that USP7 might act by removing the ubiquitin from CCDC6 protein in cells exposed to DNA damage in order to protect CCDC6 from FBXW7-mediated degradation. In our study we show that about 20% of NSCLC exhibit reduced USP7 expression and also have barely detectable levels of CCDC6. The loss of CCDC6 can cause the impairment of HR DNA repair providing the indications for the PARP inhibitors treatment in NSCLC or in tumors that harbour low levels of CCDC6 or of USP7. We wonder if the use of specific USP7 inhibitors would enhance CCDC6 turnover in order to sensitize cancer cells to the PARP inhibitors in combination with standard chemotherapies. Moreover, it has been reported that USP7 reduction is related to chemo- and radio- therapy resistance in lung adenocarcinomas [44]. Therefore, the identification of CCDC6 as a novel USP7 substrate provides the rationale for novel personalized therapy in NSCLC patients carrying USP7 deficiency.

## MATERIALS AND METHODS

### Cell lines, drugs and chemicals

HCT116 wild type, FBXW7−/− and USP7−/−, generated by Dr Bert Vogelstein and obtained by the GRCF Cell Center and BioRepository, Baltimore, MD, were grown in McCoy's 5A media (Gibco, Paisley, UK) supplemented with 10% fetal bovine serum, 1% penicillin/streptomycin, 2mM L-glutamine (Gibco, Paisley, UK) [28, 32]. The human cell lines H1975, H1299, H460, A549 were cultured in RPMI 1640 (Gibco, Paisley, UK), supplemented with 10% fetal bovine serum (Gibco, Paisley, UK), 1% penicillin/streptomycin (Gibco, Paisley, UK). No RET/PTC1 fusion or CCDC6 mutations have been reported in these cell lines [45]. HeLa Kyoto S-tag-GFP-CCDC6 cells were generated by Ina Poser in the laboratory of Anthony Hyman [23], and were grown in DMEM (high glucose), 10% FCS, 1% penicillin/streptomycin (Gibco, Paisley, UK).

Nocodazole, thymidine, SB-216763 and Cycloheximide were obtained from SIGMA-Aldrich, Inc. Deoxycytidine hydrochloride was from Fluka. MG132 and RO3306 were obtained from Calbiochem (Darmstadt, Germany). Olaparib was provided by SelleckChem (AZD2281) and cisplatinum was from SIGMA-Aldrich, Inc. Okadaic acid was from Biomol International (Farmingdale, New York).

### Sensitivity test and design for drug combination

Antiproliferative activity was determined by a modified 3-(4,5-dimethylthiazole-2-yl)-2-5-diphenyltetrazolium bromide assay, CellTiter 96 AQueous One Solution assay (Promega), as 50% inhibitory concentration (IC50) values. Briefly, cells were plated in quintuplicate in 96-well plates at a density of 1.000-3.000 cells per well, and continuously exposed to each drug for 144h. Each assay was performed in quintuplicate and IC50 values were expressed as mean +/− standard deviation. The results of the combined treatment were analyzed according to the method of Chou and Talaly by using the CalcuSyn software program [46]. The resulting combination index (CI) is a quantitative measure of the degree of interaction between different drugs. A CI value of unity denotes additive activity while CI > 1 denotes antagonism, and CI < 1 denotes synergy between agents.

### Plasmids and transfection

CCDC6 shRNA (pLKO.1 puro) was from Sigma-Aldrich. For production of stable lines, HCT116 cells were transfected with a plasmid pool (shCCDC6, NM_005436) or a pool of nontargeting vectors (sh control) by the Nucleofection. PcDNA4ToA-CCDC6 plasmids were transfected with FuGene HD (Promega) and have been described elsewhere [7]. From myc- (pcDNA4ToAmyc-his-CCDC6) template several myc-CCDC6 mutants at serine or threonine putative phosphorylation sites were created using the Quick Change Site Directed Mutagenesis Kit (Agilent, CA). To obtain the double and the triple mutant we used sequentially the single and double mutants as template with the other primers. The oligo sequences are reported in the Supplementary Table 1. Sh-FBXW7 was kindly provided by Hiroyuki Inuzuka and Wenye Wei. Ubiquitin, FBXW7, HA-FLAG-USP7 plasmids were obtained from Origene Technologies, Inc.

### Protein extract and western blot analysis

Total cell extracts were prepared with Ripa Buffer (50 mM Tris-HCl pH 7.5, 150 mM NaCl, 1% Triton X-100, 0.5% Na Deoxycholate, 0.1% SDS) and a protease inhibitor cocktail (Roche). Immunoblotting experiments were carried out according to standard procedures and visualized using the ECL chemiluminescence system (Amersham/Pharmacia Biotech).

### Real time PCR

PCR reactions were performed on RNA isolated from different cell lines using RNeasy Mini Kit (Qiagen) and reverse-transcribed using MuLV RT (Invitrogen). qRT-PCR was performed with Syber Green (Agilent) using already reported CCDC6 primers [47]. To calculate the relative expression levels we used the 2−ΔΔCT method.

### Immunofluorescence staining

Asynchronous cells were fixed with 4% paraformaldehyde and treated with phosphate-buffered saline (PBS)/0.25% Triton X-100. After staining with primary antibody, cells were washed in PBS and incubated for 30 min at room temperature with secondary antibody. Nuclei were visualized by staining with DAPI. Images were collected using a laser scanning confocal microscope (LSM 510; Carl Zeiss MicroImaging, Inc.) equipped with a plan Apo 63× NA 1.4 oil immersion objective lens. As previously described [48], the quantiﬁcation of mean ﬂuorescence intensities in selected regions of interest were performed using LSM 510 software. All z planes were volume rendered by LSM 510 software.

### Tumors

Archival tumor samples from 62 patients (males and females, smokers and nonsmokers) with NSCL cancer were retrieved from the files of the Pathology Sections of the “Ospedale Cardarelli” of Naples (N=37) and from the files of the Marshall University, Virginia, USA (N=25), with informed consent and standard IRB approvals. Clinico-pathologic data were recorded. The patient age ranged between 35 and 80 years, with a mean of 63.5 years. Patients underwent surgery between 2005 and 2010. After surgical resection, tissues were fixed in 10% neutral buffered formalin and embedded in paraffin blocks. Sections (4 μm thick) were stained with H&E. Histologic grading and pathological staging were performed according to WHO guidelines [49].

### TMA

Tissue Micro-Array (TMA) was built using the most representative areas from each single case, obtained from the Pathology Section of the “Ospedale Cardarelli” of Naples (N=37). Tissue cores with a diameter of 0.3 mm were punched from morphologically representative tissue areas of each ‘donor’ tissue block and brought into one recipient paraffin block (3×2.5 cm) using a semiautomated tissue arrayer (Galileo TMA, Milan, Italy).

### IHC analysis

For light microscopy, tissues (25 whole sections and TMA) were fixed by immersion in 10% formalin and embedded in paraffin by standard procedures; 4 μm sections were stained with haematoxylin and eosin (H&E) or processed for immunohistochemistry with anti-CCDC6 antibody, HPA-019051 (Sigma-Aldrich, Co. LLC), and anti-USP7 antibody, HPA-015641 (Sigma-Aldrich, Co. LLC). The immunohistochemical staining of USP7 and CCDC6 was evaluated semiquantitatively as the percentage of positive cells (with either nuclear or cytoplasmic localization). Cells were classified as low staining, [e.g. 0 (<5%) and + (5-25%)] and high staining [++ (26-50%) and +++ (>50%)].

### Reagents and antibodies

Anti Cyclin E (sc-247), anti Cyclin B (sc-594), anti Cyclin A (sc-751), anti Aurora A (sc-25425), anti Plk1 (sc-5585) and anti-Gsk3α/β (sc7291) were form Santa Cruz Biotechnology (CA, USA); anti-CCDC6 (ab56353) was from Abcam; anti-USP7 (A300-033A) was from Bethyl; anti-MPM2 (05-368) was from Millipore; anti-phospho-Serine (# 37430) was from Qiagen; anti-phospho-Threonine (# 9386) was from Cell Signaling Technology; anti-poly-Histidine (H1029) was from SIGMA-Aldrich, Inc. Secondary antibodies were from Biorad, California.

### Ubiquitination assay

The *in vitro* ubiquitylation assays were performed with Ubiquitinylation kit (Enzo Life Sciences). Affinity-purified GST-CCDC6 recombinant protein (Abnova H00008030-P01) was incubated with His-ubiquitin, purified E1, E2, ATP and the 293T cell lysate as source of E3 ligase FBXW7 (upon HA-FBXW7 or empty vector plasmids transient transfections). The cells were treated with MG132 (25μM) for eight hours before lysis. According to manufacturer instructions the reaction was performed at 37°C for 60 min and it was stopped by adding of 2X SDS-PAGE sample buffer. The reaction products were resolved by SDS-PAGE and probed with indicated antibodies.

## SUPPLEMENTARY MATERIAL, FIGURES, TABLES


