# Supplementary material for: FBXW7 and USP7 regulate CCDC6 turnover during the cell cycle and affect cancer drugs susceptibility in NSCLC
Source: Oncotarget. 2015 Mar 30;6(14):12697–709. doi: 10.18632/oncotarget.3708 (PMC4494967; doi:10.18632/oncotarget.3708)
Supplement: Supplementary file 1 [file oncotarget-06-12697-s001.pdf]

FBXW7 and USP7 regulate CCDC6 turnover during the cell cycle and affect cancer drugs susceptibility in NSCLC

Supplementary Material

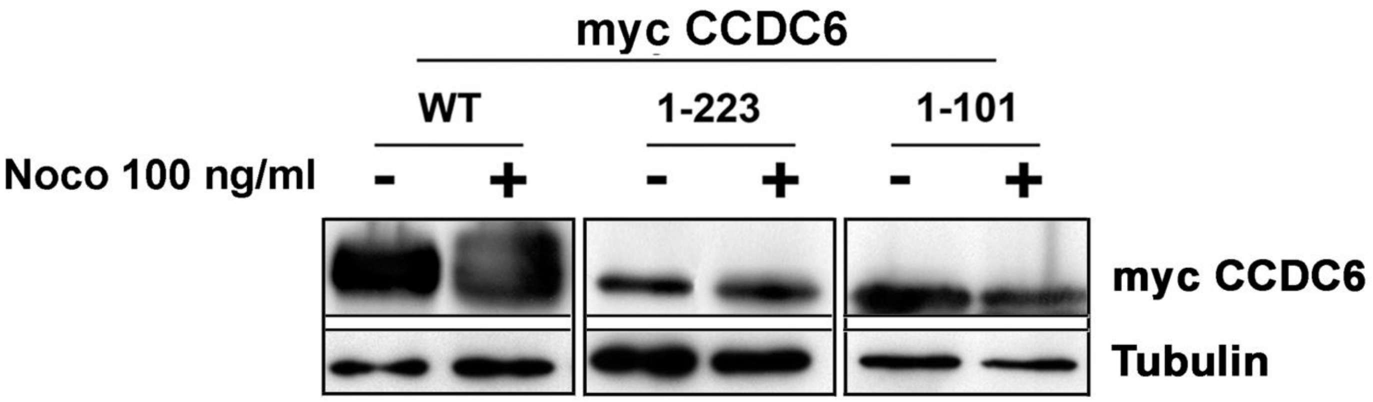

Supplementary Figure 1: Restriction of the CCDC6 mitotic phosphorylated target region

Human HeLa cells, transiently transfected with CCDC6 wild type or the CCDC6 (1-223) and (1-101) deleted mutant constructs, were treated with 100 ng/ml nocodazole for 16 hours; samples were collected and analysed by immunoblotting using the indicated antibodies.

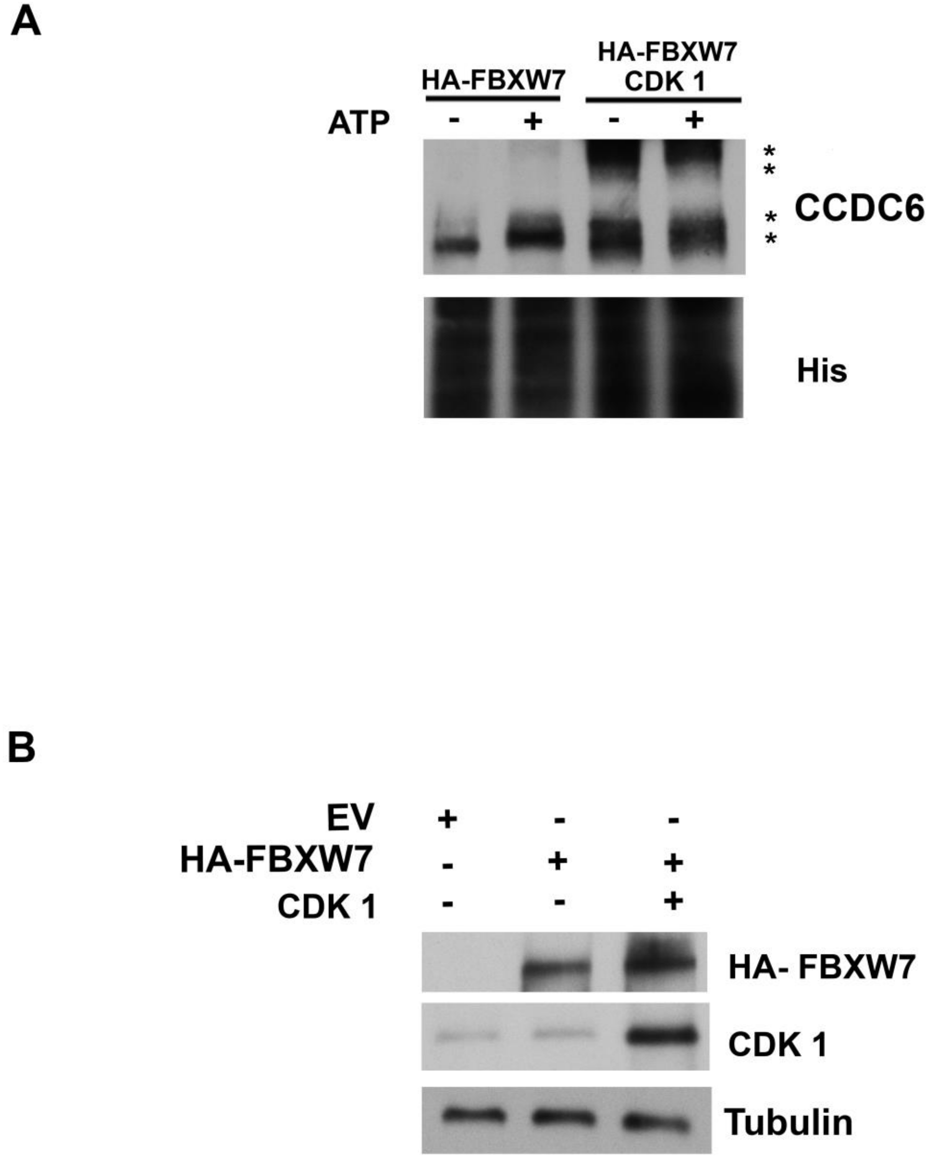

Supplementary Figure 2: CDK1 elevated FBXW7 mediated ubiquitination of recombinant GST-CCDC6 *in vitro* A) Affinity-purified GST-CCDC6 recombinant protein (Abnova H00008030-P01) was incubated with purified E1, E2, His-ubiquitin and 293T cell lysate, as source of E3 ligase FBXW7, from cells transiently transfected with HA-FBXW7, in presence or absence of CDK1 construct, at 37°C for 60 min (according to manufacturer instructions; see M&M). The ubiquitination reaction products were resolved by SDS-PAGE and probed with anti-Histidine antibody and anti-CCDC6 antibody. **B)** The transfection efficiency of the above mentioned plasmids is shown in the immunoblot hybridized with the indicated antibodies.

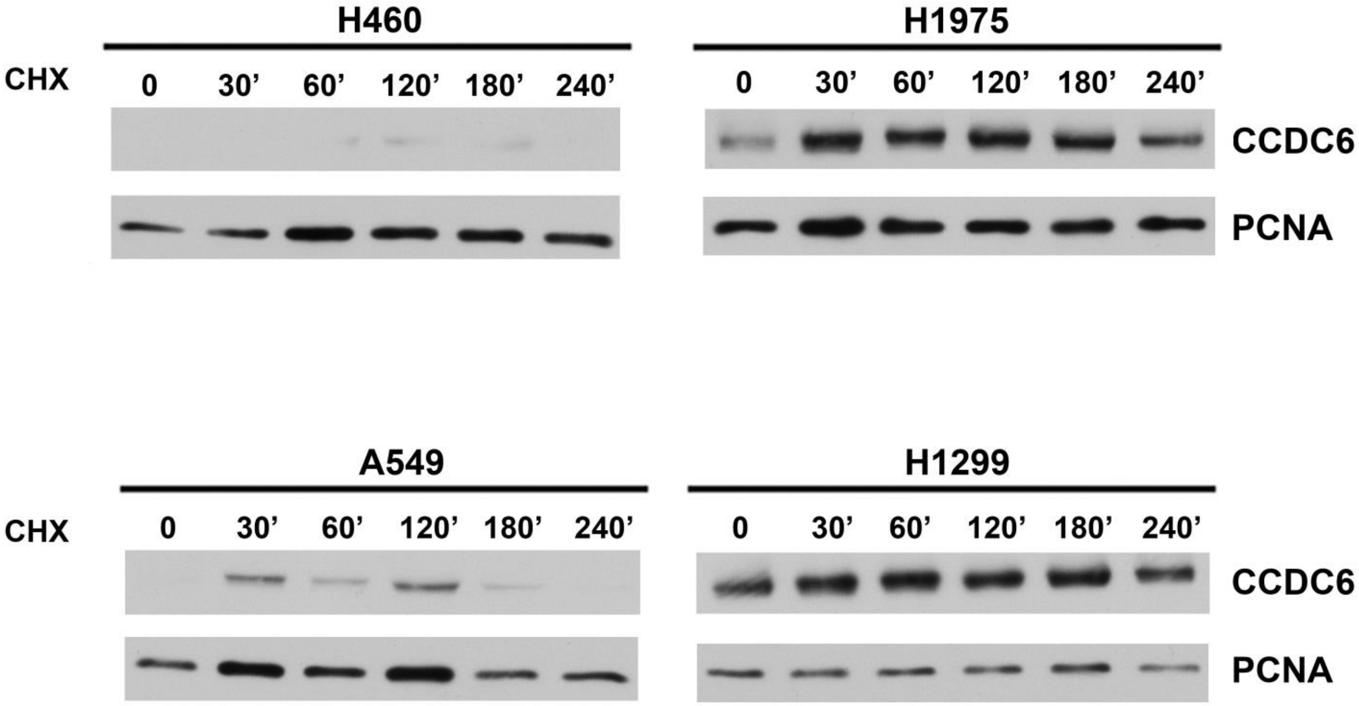

Supplementary Figure 3: In NSCLC cell lines the CCDC6 half life differences account for the observed variations in the CCDC6 abundance. H460, H1975, A549 and H1299 cells were treated with cycloheximide (CHX) (100 ng/ml) for the indicated times. Cell lysates were immunoblotted with the indicated antibodies.

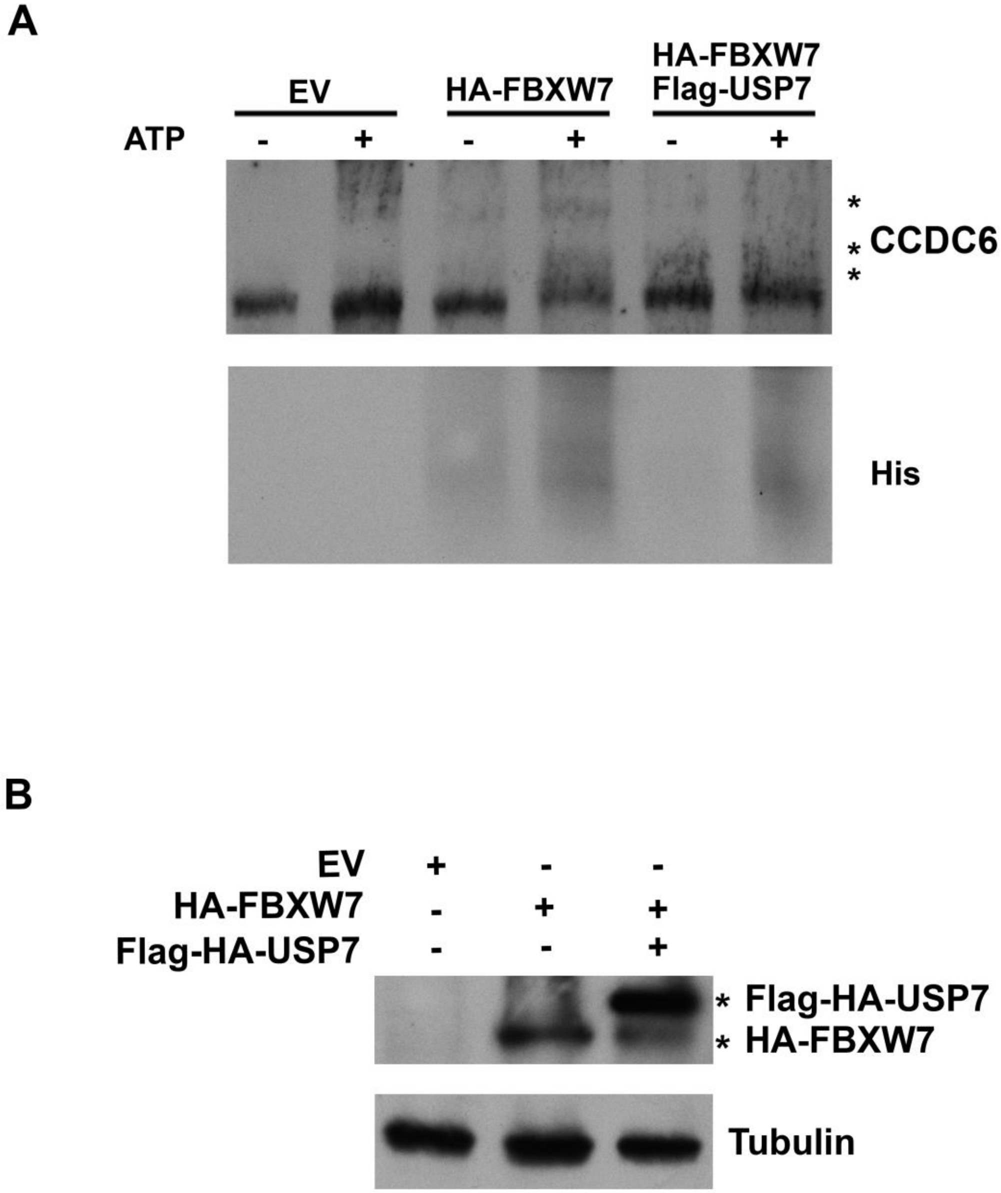

Supplementary Figure 4: USP7 impairs the CCDC6 ubiquitination mediated by FBXW7. A) Affinity-purified GST-CCDC6 recombinant protein (Abnova H00008030-P01) was incubated with purified E1, E2, His-ubiquitin and 293T cell lysate, as source of E3 ligase FBXW7, from cells transiently transfected with HA-FBXW7, in presence or absence of FLAG-HA-USP7, at 37°C for 60 min (according to manufacturer instructions; see M&M). The ubiquitination reaction products were resolved by SDS-PAGE and probed with anti-Histidine antibody and anti-CCDC6 antibody. **B)** The transfection efficiency of the above mentioned plasmids is shown in the immunoblot hybridized with the indicated antibodies.

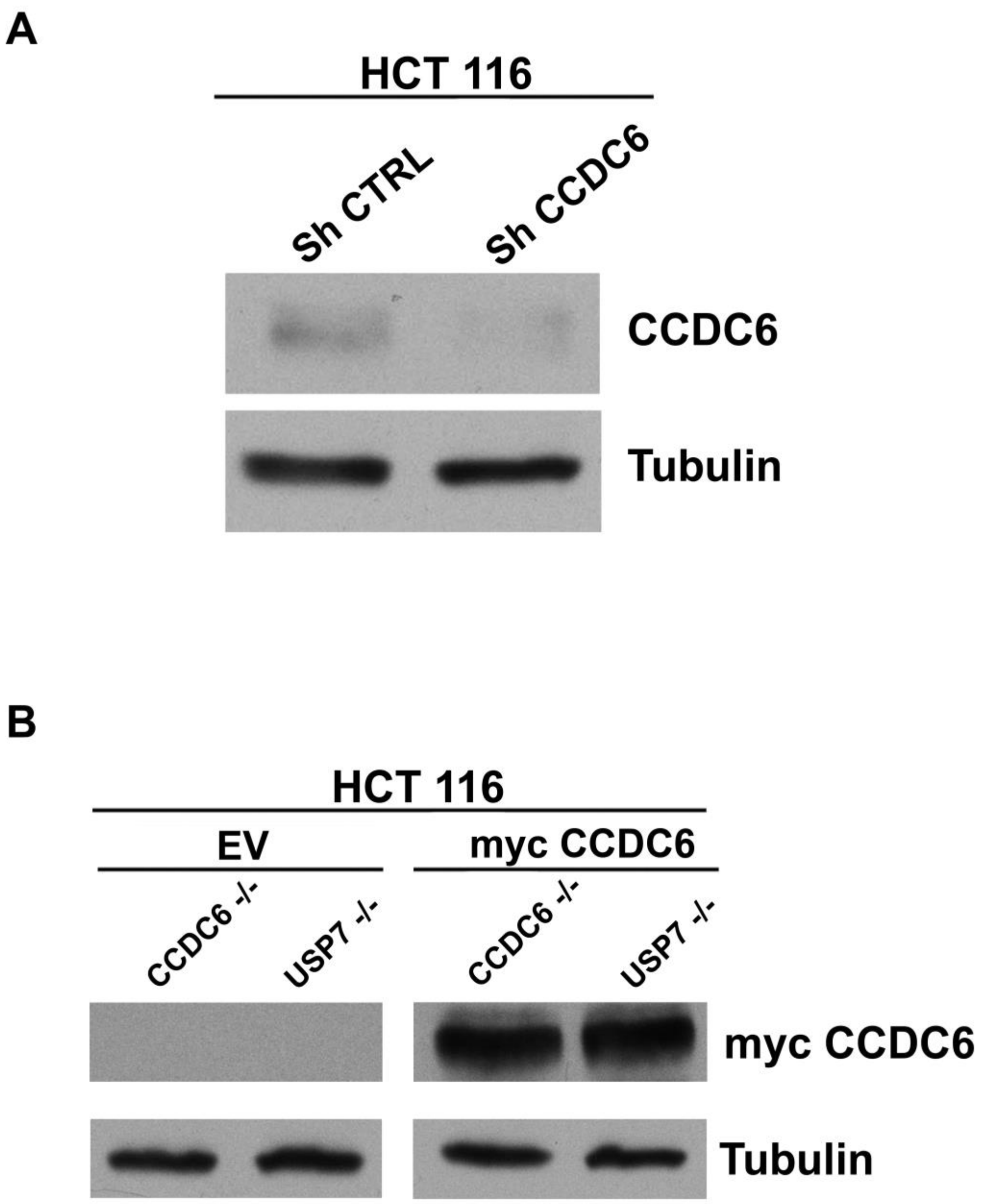

Supplementary Figure 5: A) HCT116 cells were transfected with control shRNAs (shCTRL) or sh-CCDC6. The CCDC6 protein depletion was assessed by Western blot. **B)** Myc-empty vector (EV) and myc-CCDC6 (CCDC6+) expression in HCT116 CCDC6-/- and in HCT116 USP7-/- are shown. Anti-myc hybridization is shown. Antitubulin immunoblots are shown at bottom of panels A and B, respectively.

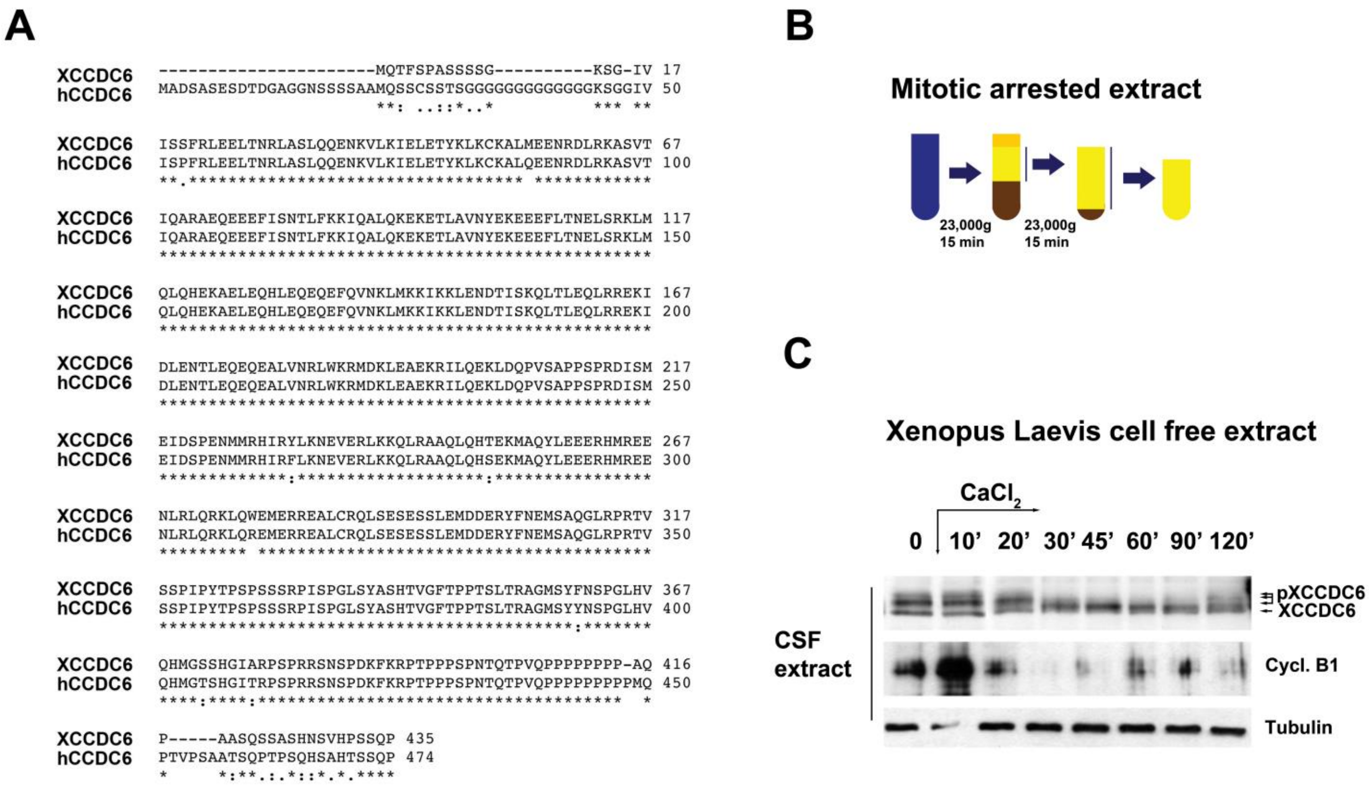

Supplementary Figure 6: CCDC6 is modified in Xenopus laevis extract. A) Xenopus CCDC6 (XCCDC6) gene product shares 94% identities with the human protein. **B)** To obtain a mitotic arrested cytosolic eggs extract, mature X. laevis females have been forced to ovulate with human chorionic gonadotropin and eggs have been collected, packed by spinning and then crushed by centrifugation. The cytoplasmic fraction has been collected and supplemented with cytochalasin and an ATP regeneration system. **C)** Post-translational modifications of XCCDC6 have been assessed during cell cycle progression by WB analysis of endogenous proteins. On the basis of the high homology we have utilized a polyclonal antibody raised against the COOH terminus of human CCDC6. Cyclin B hybridization is shown at the bottom of the figure as a control of cell cycle progression. Tubulin is shown as loading control. CSF = cytostatic factor.

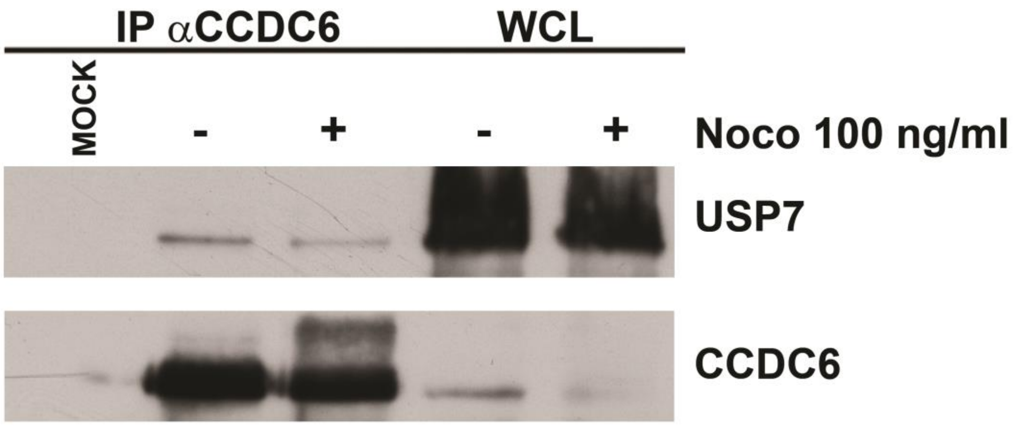

Supplementary Figure 7: USP7-CCDC6 interaction is reduced in mitosis.

Endogenous CCDC6 from asynchronous or mitotic HeLa cell extracts were analysed by SDS-PAGE and immunoblotted with the anti-USP7 and anti-CCDC6 antibodies, as shown. The immunoblots of the whole cell lysates (WCL) are shown at the right of the panel.

Supplementary Table 1:

OLIGO NAME	OLIGO SEQUENCE
$CCDC6_{S359A}$ Fwd	gatcccttacacacctgctccgagttcaagcag
$CCDC6_{S359A}$ Rev	ctgcttgaactcggagcaggtgtgtaagggatc
$CCDC6_{S413A}$ Fwd	ggtatcacaaggcctgcaccacggagaagca
$CCDC6_{S413A}$ Rev	tgcttctccgtggtgcaggccttgtgatacc
$CCDC6_{T427A}$ Fwd	gatggaggcggcgcgggccgtttgaat
$CCDC6_{T427A}$ Rev	attcaaacggcccgcgccgcctccatc
